# Energy efficiency maximization for IRS-assisted UAV short packet communication

**DOI:** 10.1038/s41598-025-92874-0

**Published:** 2025-03-17

**Authors:** Hang Hu, Senhao Zhao, Yangchao Huang, Kuanhao Yu, Qiaoyan Kang, Guobing Cheng

**Affiliations:** College of Information and Navigation, Air Force Engineering University, Xi’an, 710077 China

**Keywords:** Energy efficiency, Intelligent reflecting surface, Short packet communication, Passive beamforming, UAV trajectory, Electrical and electronic engineering, Information technology

## Abstract

With the development of the sixth generation wireless communication networks, low latency is required to support its applications. In order to meet the low latency requirement, short packet communication is considered to be used, in which a ground sensor transmits the sensing information to a fixed-wing unmanned aerial vehicle (UAV). In this paper, we consider maximizing the energy efficiency of intelligent reflecting surface (IRS)-assisted UAV short packet communication by optimizing the UAV’s speed, trajectory, transmit power and passive beamforming of IRS. Since the maximization problem is nonconvex with respect to the system parameters, this problem is difficult to be solved. Therefore, the successive convex approximation method is employed and a joint iterative optimization algorithm is proposed to solve this problem. In the simulation parts, it is shown that the algorithm proposed in this paper has good convergence performance. And there exists an optimal value of flight speed for the UAV to minimize the energy consumption. In addition, it is found that the application of IRS can improve the energy efficiency effectively.

## Introduction

With development of communication technology, 6G wireless network is beginning to be widely studied^1–3^. Based on the improvement of the fifth generation wireless networks, the 6G wireless networks will enable complete digitization and interconnection of everything, and will promote the realization of various applications, such as eHealth, industry 5.0, connected robots and autonomous systems, and wireless sensor networks^4–8^. The unmanned aerial vehicle (UAV) plays an important role in air-to-ground communication networks. In^9^, the energy consumption of the user equipment is minimized in a UAV assisted mobile edge computing (MEC) system. In^10^, the trajectory design and fairness for facilitating ultra-reliable and low latency communication (URLLC) in UAV-enabled MEC system is investigated.

In order to satisfy the demanding latency requirements of future 6G wireless networks, short packet communication (SPC) is considered^11,12^. In short packet communication, the probability of decoding errors cannot be ignored due to the use of finite blocklength coding^13,14^. In^15^, the authors investigated the transmission rate for short packet communication, and it provides a basis for the study of SPC.

Due to the flexible deployment of the UAV, it is applied widely in short packet communication^16–19^. The authors in^20^ derived an expression for the average information age of the UAV-assisted SPC based on a stochastic hybrid model. An optimization method is proposed in^21^ to enhance the reliability of UAV-assisted visible light communication with finite blocklength coding. In^22^, the throughput and reliability of UAV communication systems based on nonlinear energy harvesting are analyzed. Specifically, the finite blocklength coding and infinite blocklength coding are studied respectively. In^23^, the maximization of energy efficiency for UAV relay SPC scenario is studied. In^24^, the UAV short packet communication process is divided into two phases, one is data collection and the other is data transmission. And the energy efficiency optimization in data collection and the secure rate optimization in data transmission are studied respectively. In summary, optimizing the performance of UAV short packet communication is a new challenge in future IoT networks.

With the development of the city, the density and height of buildings are increasing. It is possible that the data link between the ground sensors and the UAV are obscured by buildings. Then, some researchers proposed intelligent reflecting surface (IRS) to improve the channel quality and the communication performance. By reflecting the transmitted signal and adjusting the IRS’s phase shifts, the received signals from different paths can be enhanced^25^. For the passive IRS, it is controlled by low power electronic circuits, and the power consumption is nearly zero^26^. The phase shift of the linear IRS can be adjusted to increase the energy of the signal and improve the communication rate^27^. And linear IRS can adjust its reflection characteristics according to real-time communication requirements, which can adapt to different transmission environments. In^28^, the UAV-mounted IRS for data collection is investigated in an energy-aware manner. And the Synergetic UAV-IRS communication system is considered in^29^, the directional antenna is used to improve the system performance. In^30^, the data rate is maximized by optimizing the UAV’s trajectory, user scheduling with mobility and power consumption constraints. In^31^, the authors study the secure communication of UAV downlink communication systems with assistance of IRS. In^32^, the existence of jamming in UAV communication with IRS is considered, and the energy efficiency is optimized. The UAV in^33^ is used as an aerial relay to transmit information to the base station (BS), and the eavesdroppers are considered. The authors in^34^ studied the maximization of energy efficiency and spectrum efficiency in UAV communication systems with assistance of IRS. In^35^, the UAV downlink communication with IRS is considered, the sum transmission rate is improved by optimizing the channel resource allocation, UAV placement and beamforming. The research on improving efficiency of IRS will accelerate the realization of 6G communications and green communications.

The above studies on IRS-assisted UAV communication have not considered short packet communication. With development of Internet of Things (IoT), the performance optimization of IRS-assisted UAV SPC has become an urgent research topic^36^. In recent years, some researchers have carried out research work on UAV SPC systems with assistance of IRS. In^37^, the UAV and IRS are used as relay nodes for multi-hop relay communication, and the authors improve the communication reliability by optimizing blocklength allocation, UAV’s position and the phase shift of IRS. In^38^, multiple IRS-equipped UAVs are considered as relays to transmit data from the base station to multiple user groups, and the probability of decoding error is minimized by optimizing the deployment of UAV, beamforming and blocklength allocation. In^39^, the authors considered a scenario where an access point sends short packet information to the receivers with UAV-mounted IRS. The maximum average age of information is minimized by optimizing the time interval allocation, power allocation and trajectory of UAV.

In IRS-assisted UAV short packet communication, the battery energy carried by the small UAV is limited. However, in most existing studies, the energy efficiency optimization for IRS-assisted UAV SPC is not considered. In this paper, the energy efficiency maximization for UAV short packet communication with assistance of IRS is studied, in which the ground sensor transmits its sensing information to a fixed-wing UAV. The main contributions are listed as follows.

(1) The system model of UAV short packet communication with assistance of IRS is presented in detail. And the mathematical expression of the energy efficiency maximization problem for UAV SPC system with IRS is formulated. The energy efficiency is considered to be maximized by optimizing the passive beamforming of IRS, UAV’s speed, trajectory and transmit power.

(2) The problem of energy efficiency maximization is nonconvex with respect to the system parameters, hence it is difficult to be solved. In order to obtain the optimal solutions, the original optimization problem is firstly divided into three subproblems, then the subproblems are solved separately, and finally the problem is solved by employing an efficient iterative optimization algorithm.

(3) In the numerical results, it is seen that the proposed scheme can obtain higher performance of energy efficiency than benchmark schemes. And there exists an optimal value of flight speed for the UAV to minimize its power consumption. Comparing the scheme without IRS, it can be found that the application of IRS can improve the energy efficiency effectively.

The remaining sections of this paper are organized as follows. In section “System model and problem formulation”, we present the IRS-assisted UAV short packet communication model and formulate the optimization problem. Section “Optimal solutions” is devoted to solve the energy efficiency maximization problem. Simulation results are shown in section “Numerical results”. The conclusion is given in section “Conclusions”.

## System model and problem formulation

**Fig. 1:**
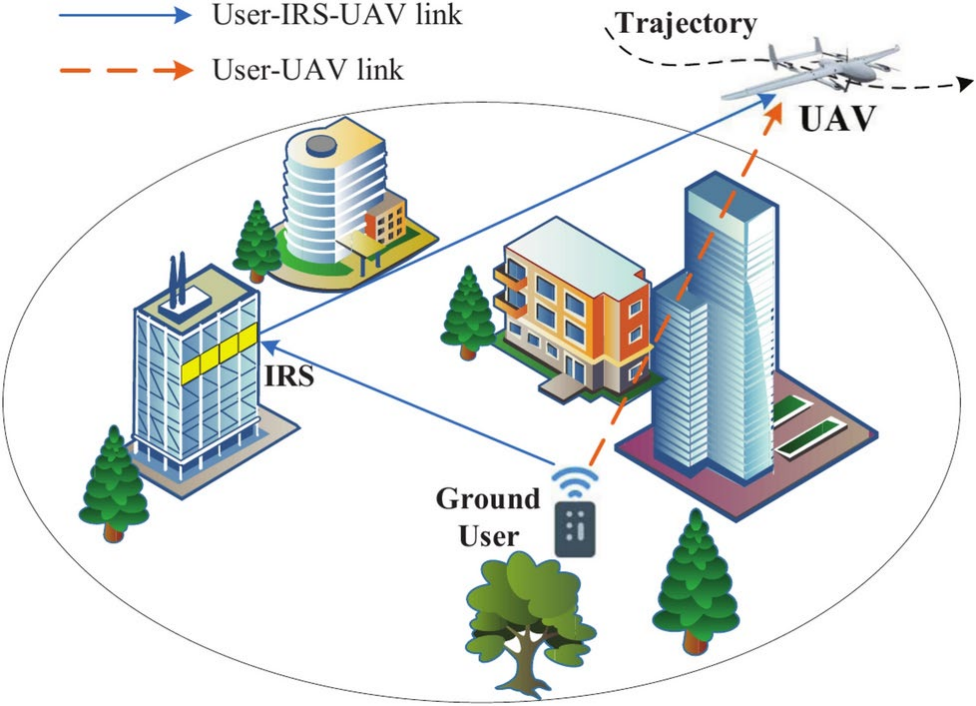
UAV short packet communication system with assistance of IRS.

The system model is shown in Fig. 1, in which a ground sensor *d* transmits its sensing information to a fixed-wing UAV. Unlike conventional communications, we assume that the ground sensor transmits short packet messages. Since the transmission link from the ground sensor to the UAV is obscured due to the presence of high buildings, IRS is employed to enhance the wireless channel quality. The UAV is flying with altitude $z_u.$ For the convenience of analysis, the UAV’s flight time $\text {T}$ is divided equally into $\text {M}$ slots, where each slot is $\delta _t=\text {T}/\text {M}.$ Then, at time slot *n*, the UAV’s speed is denoted as ${\mathbf{v}}[n]=(v_x[n],v_y[n]),$ and its horizontal position is ${\mathbf{Q}}[n]=(x[n],y[n]),n\in [1,\text {M}].$ The horizontal positions of the ground sensor *d* and the IRS are denoted as ${\mathbf{C}}_d=(x_d,y_d),$ ${\mathbf{C}}_r=(x_r,y_r),$ respectively. The altitude of the ground sensor *d* is $z_d,$ and the altitude of the IRS is $z_r.$

Due to the obstruction of urban buildings, the signal transmitted by the ground sensor *d* reaches the UAV with multiple reflections and scattering. The wireless channel from ground sensor *d* to the UAV can be considered as Rayleigh fading channel, and the corresponding channel gain is

1 $$\begin{aligned} h_{du}[n]=\sqrt{\alpha \text {D}_{du}^{-\beta _{du}}[n]}{\tilde{h}}_{du}[n], \end{aligned}$$

where $\alpha$ is channel power gain with distance of 1m, $\beta _{du}$ is the path loss exponent of the transmission link from ground sensor *d* to the UAV, $\text {D}_{du}[n]$ is the distance between the ground sensor *d* and the UAV, ${\tilde{h}}_{du}[n]$ follows circularly symmetric complex Gaussian (CSCG) distribution.

The IRS is composed of $\text {L}$ reflecting elements. To improve the transmission performance, the IRS reflects the signal sent by the ground sensor *d*. The phase shift matrix of IRS is given as follows

2 $$\begin{aligned} \varvec{\Psi }[n]=\text {diag}\Big (e^{j\psi _1[n]},e^{j\psi _2[n]},...,e^{j\psi _l[n]},...,e^{j\psi _\text {L}[n]}\Big ),l\in [1,\text {L}] \end{aligned}$$

where $\psi _l[n]$ denotes the phase shift of *l*th reflecting element.

The channel from the ground sensor *d* to IRS consists of LoS and NLoS components. Therefore, it is considered as a Rician fading channel. The channel gain from the ground sensor *d* to the IRS is expressed as

3 $$\begin{aligned} {\mathbf{h}}_{dr}=\sqrt{\alpha \text {D}_{dr}^{-\beta _{dr}}}\tilde{{\mathbf{h}}}_{dr}\in {\mathbb {C}}^{\text {L}\times 1}, \end{aligned}$$

where $\text {D}_{dr}$ is the distance between the ground sensor *d* and the IRS, $\beta _{dr}$ is the path loss exponent of the transmission link from ground sensor *d* to the IRS, $\tilde{{\mathbf{h}}}_{dr}$ denotes the small-scale fading component. Specifically, the expression of $\tilde{{\mathbf{h}}}_{dr}$ is

4 $$\begin{aligned} \tilde{{\mathbf{h}}}_{dr}=\sqrt{\frac{\kappa }{\kappa +1}}\tilde{{\mathbf{h}}}_{dr}^\text {L}+\sqrt{\frac{1}{\kappa +1}}\tilde{{\mathbf{h}}}_{dr}^\text {N} \in {\mathbb {C}}^{\text {L}\times 1}, \end{aligned}$$

where $\kappa$ is Rician fading factor, $\tilde{{\mathbf{h}}}_{dr}^\text {L}$ is the array response of the IRS to the signal transmitted by the ground sensor *d*, $\tilde{{\mathbf{h}}}_{dr}^\text {N}$ is the NLoS component of the link from the ground sensor *d* to the IRS, which follows CSCG distribution. When the positions of the ground sensor *d* and the IRS are known, the expression of $\tilde{{\mathbf{h}}}_{dr}^\text {L}$ is

5 $$\begin{aligned} \tilde{{\mathbf{h}}}_{dr}^\text {L}=\Big (1,e^{-j\frac{2\pi }{\lambda }\phi \cos \theta _{dr}},...,e^{-j\frac{2\pi }{\lambda }\phi (\text {L}-1)\cos \theta _{dr}}\Big )^\text {T}, \end{aligned}$$

where $\phi$ is the reflecting element spacing of IRS, $\lambda$ is the carrier wavelength transmitted by ground sensor *d*, $\theta _{dr}$ is the arrival angle of the link from the ground sensor *d* to the IRS, and $\cos \theta _{dr}=(x_d-x_r)/{\text {D}_{dr}}.$

Since the UAV and the IRS is far from the ground and is usually not obscured by high objects such as buildings and trees, the link from the IRS to the UAV is considered as LoS link. Therefore, the corresponding channel gain is

6 $$\begin{aligned} {\mathbf{h}}_{ru}[n]=\sqrt{\alpha \text {D}_{ru}^{-2}[n]}\tilde{{\mathbf{h}}}_{ru}[n] \in {\mathbb {C}}^{\text {L}\times 1}, \end{aligned}$$

where $\text {D}_{ru}[n]$ is the distance between IRS and UAV, $\tilde{{\mathbf{h}}}_{ru}[n]$ is the array response. When the positions of the IRS and the UAV are known, the expression of $\tilde{{\mathbf{h}}}_{ru}[n]$ is obtained as follows

7 $$\begin{aligned} \tilde{{\mathbf{h}}}_{ru}[n]=\Big (1,e^{-j\frac{2\pi }{\lambda }\phi \cos \theta _{ru}[n]},...,e^{-j\frac{2\pi }{\lambda }\phi (\text {L}-1)\cos \theta _{ru}[n]}\Big )^\text {T}, \end{aligned}$$

where $\theta _{ru}[n]$ is the departure angle of the transmission link from the IRS to the UAV, $\cos \theta _{ru}[n]=(x_r-x_u[n])/{\text {D}_{ru}[n]}.$ According to the current state of research in channel estimation techniques^40–42^, the channel state information (CSI) for IRS-assisted communication systems can be obtained.

The signal received by the UAV has two components, one is the signal transmitted by the ground sensor, and the other is the signal reflected by the IRS. According to the above analysis, it can be obtained that the channel gain from the ground sensor *d* to the UAV (reflected by IRS) is ${\mathbf{h}}_{ru}^{\text {H}}\varvec{\Psi }[n]{\mathbf{h}}_{dr}.$ Then, the signal-to-noise ratio (SNR) of received signal at UAV is

8 $$\begin{aligned} \gamma [n]=\frac{\text {P}[n]\big |h_{du}[n]+{\mathbf{h}}_{ru}^\text {H}[n]\varvec{\Psi }[n]{\mathbf{h}}_{dr}\big |^2}{\sigma ^2}, \end{aligned}$$

where $\text {P}[n]$ is the ground sensor’s transmit power, $\sigma ^2$ is the variance of AWGN.

For short packet communication, according to^15^, the maximum transmission rate at the UAV is given as follows

9 $$\begin{aligned} \text {R}[n]=\log _2(1+\gamma [n])-\sqrt{\frac{\text {V}[n]}{m}}\frac{{Q}^{-1}(\epsilon )}{\ln 2}, \end{aligned}$$

where $\text {V}[n]=1-(1+\gamma [n])^{-2}$ is the channel dispersion, $\epsilon$ is the decoding error probability of the system, *m* is the blocklength.

Since the energy of the UAV is limited, we consider maximizing the energy efficiency of the UAV SPC system. Therefore, the energy consumed by the UAV during time period $\text {T}$ needs to be studied. Compared to the energy consumed for UAV flight, the energy consumed for communication can be negligible. Based on the analysis in^43^, the energy consumption of the UAV flight during time period $\text {T}$ is

10 $$\begin{aligned} \begin{aligned} \text {E}^{\text {total}}=\delta _t\sum _{n=1}^{\text {M}}\Bigg [\frac{1}{2}s_0\Arrowvert {\mathbf{v}}[n]\Vert ^3+\text {P}_1\Big (1+\frac{3\Vert {\mathbf{v}}[n]\Vert ^2}{\varpi ^2}\Big )+\text {P}_2\Bigg (\sqrt{1+\frac{\Vert {\mathbf{v}}[n]\Vert ^4}{4\text {U}_0^4}}-\frac{\Vert {\mathbf{v}}[n]\Vert ^2}{2\text {U}_0^2}\Bigg )^{1/2}\Bigg ], \end{aligned} \end{aligned}$$

where $\text {P}_1$ and $\text {P}_2$ are the power parameters in the hovering state of the UAV, $s_0$ is the parameter related to the parasite power, $\varpi$ and $\text {U}_0$ are the parameters related to the rotor blade velocity. In this paper, the energy efficiency is the ratio of the average transmitted bits over the average energy consumption, i.e., $\Upsilon =(\sum _{n=1}^{\text {M}}\text {R}[n]\delta _t)/\text {E}^{\text {total}}.$

Next, we will maximize the energy efficiency by optimizing the ground sensor’s transmit power ${\mathbf{P}}=\{{\text {P}[n], \forall n}\},$ the IRS’s passive beamforming $\varvec{\Psi }=\{{\varvec{\Psi }[n], \forall n}\},$ the UAV’s speed ${\mathbf{v}}=\{{{\mathbf{v}}[n], \forall n}\}$ and trajectory ${\mathbf{Q}}=\{{{\mathbf{Q}}[n], \forall n}\}.$ Specifically, we formulate the optimization problem as follows

11a $$\begin{aligned} \text{(OP): }&\;\;\mathop {\max }\limits _{{\mathbf{P}},\varvec{\Psi },{\mathbf{Q}},{\mathbf{v}}} \,\, \frac{\sum _{n=1}^\text {M} \text {R}[n]\delta _t}{\text {E}^{\text {total}}} \end{aligned}$$

11b $$\begin{aligned} \text{ s.t.: }&\;\; {\mathbf{Q}}[n]={\mathbf{Q}}[n-1]+{\mathbf{v}}[n-1]\delta _t,n=2,...,\text {M}\end{aligned}$$

11c $$\begin{aligned}&\;\; {\mathbf{Q}}[1]={\mathbf{Q}}_\text {I} \end{aligned}$$

11d $$\begin{aligned}&\;\; {\mathbf{Q}}[\text {M}]+{\mathbf{v}}[\text {M}]\delta _t={\mathbf{Q}}_\text {F} \end{aligned}$$

11e $$\begin{aligned}&\;\; \Vert {\mathbf{v}}[n]\Vert \le v_{\max },\forall {n} \end{aligned}$$

11f $$\begin{aligned}&\;\; 0 \le \text {P}[n]\le \text {P}_{\max } \end{aligned}$$

11g $$\begin{aligned}&\;\; \frac{1}{\text {M}}\sum _{n=1}^\text {M} \text {P}[n]\le \text {P}_{\text {avg}} \end{aligned}$$

11h $$\begin{aligned}&\;\; 0\le \psi _l[n]\le 2\pi , \forall l,n \end{aligned}$$

where ${\mathbf{Q}}_\text {I}$ is the initial position of the UAV and ${\mathbf{Q}}_\text {F}$ is the final position, constraints (11b)–(11d) are the trajectory constraints, (11e) is the constraint of UAV’s speed and $v_{\max }$ is the UAV’s maximum speed, (11g) is the constraint of UAV’s transmit power, $\text {P}_{\max }$ and $\text {P}_{\text {avg}}$ are the maximum transmit power and the average transmit power of ground sensor *d*, respectively, and constraint (11h) specifies the phase shift range of IRS.

## Optimal solutions

The optimization problem (11) can be transformed to problem (12) equivalently by introducing two auxiliary variables $\varvec{\zeta }=\{\zeta [n],\forall n\}$ and $\varvec{\eta }=\{\eta [n],\forall n\},$ which is rewritten as

12a $$\begin{aligned} \text{(OP1): }&\;\;\mathop {\max }\limits _{{\mathbf{P}},\varvec{\Psi },{\mathbf{Q}},{\mathbf{v}},\varvec{\zeta },\varvec{\eta }} \,\, \frac{\sum _{n=1}^\text {M} \tilde{\text {R}}[n]\delta _t}{\text {E}^{\text {total}}} \end{aligned}$$

12b $$\begin{aligned} \text{ s.t.: }&\;\; (11\text {b})-(11\text {h})\end{aligned}$$

12c $$\begin{aligned}&\;\; \zeta [n]\ge 1-(1+\eta [n])^{-2},\forall n \end{aligned}$$

12d $$\begin{aligned}&\;\; \eta [n]\ge \frac{\text {P}[n]|h_{du}[n]+{\mathbf{h}}_{ru}^\text {H}\varvec{\Psi }[n]{\mathbf{h}}_{dr}|^2}{\sigma ^2},\forall n \end{aligned}$$

where

13 $$\begin{aligned} \tilde{\text {R}}[n]=\log _2(1+\gamma [n])-\sqrt{\frac{\zeta [n]}{m}}\frac{{Q}^{-1}(\epsilon )}{\ln 2}. \end{aligned}$$

The above problem is difficult to be solved since it is nonconvex. To solve this difficulty, problem (12) is firstly divided into three subproblems, then the subproblems are solved respectively, and finally an iterative algorithm will be proposed.

### Transmit power optimization

Given the IRS’s passive beamforming $\varvec{\Psi }=\{\varvec{\Psi }[n],\forall n\},$ the UAV’s speed ${\mathbf{v}}=\{{\mathbf{v}}[n],\forall n\}$ and trajectory ${\mathbf{Q}}=\{{\mathbf{Q}}[n],\forall n\},$ we firstly optimize the transmit power of the ground sensor *d*. Problem (12) is rewritten as

14a $$\begin{aligned} \text{(OP1.1): }&\mathop {\max }\limits _{{\mathbf{P}},\varvec{\zeta },\varvec{\eta }} \,\, {\sum _{n=1}^\text {M} \tilde{\text {R}}[n]\delta _t} \end{aligned}$$

14b $$\begin{aligned} \text{ s.t.: }&(11\text {f}),(11\text {g}),(12\text {c}),(12\text {d}) \end{aligned}$$

It can be obtained that problem (14) is nonconvex. We will address this difficulty by using SCA methods.

By using the first order Taylor expansion method, we derive the concave lower bound function of $\tilde{\text {R}}[n]$ as follows

15 $$\begin{aligned} \begin{aligned}&\tilde{\text {R}}[n]=\log _2(1+\gamma [n])-\sqrt{\frac{\zeta [n]}{m}}\frac{{Q}^{-1}(\epsilon )}{\ln 2}\\&\ge \log _2(1+\gamma [n])-\Big \{\zeta _0^{1/2}[n]+\zeta [n]\zeta _0^{-1/2}[n]\Big \}\frac{{Q}^{-1}(\epsilon )}{2\sqrt{m}\ln 2}\\&=\tilde{\text {R}}_p^{lb}[n], \end{aligned} \end{aligned}$$

where $\zeta _0[n]$ is a given feasible point of $\zeta [n]$.

By using the SCA method, constraint (12c) can be equivalently replaced by

16 $$\begin{aligned} \begin{aligned} \zeta [n]\ge 1-(1+\eta _0[n])^{-2}+2(1+\eta _0[n])^{-3}(\eta [n]-\eta _0[n]),\forall n \end{aligned} \end{aligned}$$

Based on the above analysis, problem (14) will be reformulated as

17a $$\begin{aligned} \text{(OP1.2): }&\mathop {\max }\limits _{{\mathbf{P}},\varvec{\zeta },\varvec{\eta }} \,\, {\sum _{n=1}^\text {M} \tilde{\text {R}}_p^{lb}[n]\delta _t} \end{aligned}$$

17b $$\begin{aligned} \text{ s.t.: }&(11\text {f}),(11\text {g}),(12\text {d}),(16) \end{aligned}$$

Problem (17) is convex, and we can use CVX to solve it.

### Passive beamforming optimization

Given ground sensor’s transmit power ${\mathbf{P}}=\{\text {P}[n],\forall n\},$ UAV’s speed ${\mathbf{v}}=\{{\mathbf{v}}[n],\forall n\}$ and trajectory ${\mathbf{Q}}=\{{\mathbf{Q}}[n],\forall n\},$ we optimize passive beamforming of the IRS. Problem (12) is reformulated as

18a $$\begin{aligned} \text{(OP2.1): }&\mathop {\max }\limits _{\varvec{\Psi },\varvec{\zeta },\varvec{\eta }} \,\, {\sum _{n=1}^\text {M} \tilde{\text {R}}[n]\delta _t} \end{aligned}$$

18b $$\begin{aligned} \text{ s.t.: }&(11\text {h}),(12\text {c}),(12\text {d}) \end{aligned}$$

We define

19 $$\begin{aligned} {\mathbf{H}}_u[n]= & \Big [\tilde{{\mathbf{h}}}_{ru}^\text {H}[n],{\tilde{h}}_{du}[n]\Big ], \end{aligned}$$

20 $$\begin{aligned} {\mathbf{G}}[n]= & \text {diag}\Bigg [\sqrt{\alpha \text {D}_{ru}^{-2}[n]}{\mathbf{h}}_{dr}^\text {T},\sqrt{\alpha \text {D}_{du}^{-\beta _{du}}[n]}\Bigg ], \end{aligned}$$

21 $$\begin{aligned} {\mathbf{z}}[n]= & [z_1[n],z_2[n],...,z_\text {L}[n],1]^\text {T}, \end{aligned}$$

where $$z_l[n]=e^{j\psi _l[n]},l\in [1,\text {L}]$$.

According to (19)–(21), we can obtain the equation as follows

22 $$\begin{aligned} h_{du}[n]+{\mathbf{h}}_{ru}^\text {H}[n]\varvec{\Psi }[n]{\mathbf{h}}_{dr}={\mathbf{H}}_u[n]{\mathbf{G}}[n]{\mathbf{z}}[n]. \end{aligned}$$

Thus, problem (18) can be rewritten as

23a $$\begin{aligned} \text{(OP2.2): }&\mathop {\max }\limits _{\mathbf{z},\varvec{\zeta },\varvec{\eta }} \,\, \delta _t{\sum _{n=1}^\text {M} \log _2\bigg (1+\frac{\text {P}[n]|\mathbf{H}_u[n]\mathbf{G}[n]\mathbf{z}[n]|^2}{\sigma ^2}\bigg )}-\sqrt{\frac{\zeta [n]}{m}}\frac{{Q}^{-1}(\epsilon )}{\ln 2} \nonumber \\ \text{ s.t.: }&\zeta [n]\ge 1-(1+\eta [n])^{-2},\forall n \end{aligned}$$

23b $$\begin{aligned}&\eta [n]\ge \frac{\text {P}[n]|\mathbf{H}_u[n]\mathbf{G}[n]\mathbf{z}[n]|^2}{\sigma ^2},\forall n \end{aligned}$$

23c $$\begin{aligned}&\big |z_l[n]\big |=1,l=1,...,\text {L},\forall n \end{aligned}$$

Since the objective function is nonconcave and the constraints (23b) are nonconvex, problem (23) is nonconvex. Firstly, we employ the same method as in the previous section to transform constraint (23b) into constraint (16). Then, $|{\mathbf{H}}_u[n]{\mathbf{G}}[n]{\mathbf{z}}[n]|^2$ can be replaced as

24 $$\begin{aligned} \begin{aligned} |{\mathbf{H}}_u[n]{\mathbf{G}}[n]{\mathbf{z}}[n]|^2&={\mathbf{H}}_u[n]{\mathbf{G}}[n]{\mathbf{z}}[n]{\mathbf{z}}^\text {H}[n]{\mathbf{G}}^\text {H}[n]{\mathbf{H}}_u^\text {H}[n]\\&=\text {Tr}\Big ({\mathbf{S}}[n]{\mathbf{z}}[n]{\mathbf{z}}^\text {H}[n]\Big ), \end{aligned} \end{aligned}$$

where $${\mathbf{S}}[n]={\mathbf{G}}^\text {H}[n]{\mathbf{H}}_u^\text {H}[n]{\mathbf{H}}_u[n]{\mathbf{G}}[n].$$

When the rank of ${\mathbf{Z}}[n]$ is one and ${\mathbf{Z}}[n]\succeq 0,$ we obtain

25 $$\begin{aligned} {\mathbf{Z}}[n]={\mathbf{z}}[n]{\mathbf{z}}^\text {H}[n]. \end{aligned}$$

Given the point $\zeta _0,$ we obtain the concave lower bound of $\tilde{\text {R}}[n]$ as follows

26 $$\begin{aligned} \begin{aligned} \tilde{\text {R}}[n]&=\log _2(1+\frac{\text {P}[n]\text {Tr}({\mathbf{S}}[n]{\mathbf{Z}}[n])}{\sigma ^2})-\sqrt{\frac{\zeta [n]}{m}}\frac{{Q}^{-1}(\epsilon )}{\ln 2}\\&\ge \log _2(1+\frac{\text {P}[n]\text {Tr}({\mathbf{S}}[n]{\mathbf{Z}}[n])}{\sigma ^2})-\Big \{\zeta _0^{1/2}[n]+\zeta [n]\zeta _0^{-1/2}[n]\Big \}\frac{{Q}^{-1}(\epsilon )}{2\sqrt{m}\ln 2}\\&=\tilde{\text {R}}_{\psi }^{lb}[n]. \end{aligned} \end{aligned}$$

To address that $\text {Rank}({\mathbf{Z}}[n])=1$ is nonconvex, we use semidefinite relaxation method. Therefore, the problem (23) is reformulated as follows

27a $$\begin{aligned} \text{(OP2.3): }&\mathop {\max }\limits _{{\mathbf{z}},\varvec{\zeta },\varvec{\eta }} \,\, \delta _t\sum _{n=1}^\text {M}\tilde{\text {R}}_{\psi }^{lb}[n] \end{aligned}$$

27b $$\begin{aligned} \text{ s.t.: }&\zeta [n]\ge 1-(1+\eta _0[n])^{-2}+2(1+\eta _0[n])^{-3}(\eta [n]-\eta _0[n]),\forall n \nonumber \\&\eta [n]\ge \frac{\text {P}[n]\text {Tr}({\mathbf{S}}[n]{\mathbf{Z}}[n])}{\sigma ^2},\forall n \end{aligned}$$

27c $$\begin{aligned}&|z_l[n]|=1,\forall l,n \end{aligned}$$

27d $$\begin{aligned}&{\mathbf{Z}}[n]\succeq 0,\forall n \end{aligned}$$

where ${\mathbf{Z}}=\{{\mathbf{Z}}[n],\forall n\}.$ The problem (27) is convex, so we can use CVX toolbox to solve it. Because of the relaxation of constraint $\text {Rank}({\mathbf{Z}}[n])=1,$ solution of problem (27) is an upper bound of the solution of problem (18). After solving problem (27), we will find a solution that satisfies constraint $\text {Rank}({\mathbf{Z}}[n])=1.$ Firstly, we perform an eigenvalue decomposition of ${\mathbf{Z}}[n]$ to obtain ${\mathbf{Z}}[n]={\mathbf{F}}[n]{\mathbf{M}}[n]{\mathbf{F}}^\text {H}[n].$ Then we define $\bar{{\mathbf{z}}}[n]={\mathbf{F}}[n]{\mathbf{M}}^{1/2}[n]{\mathbf{r}}[n],$ where ${\mathbf{r}}[n]$ follows CSCG distribution. Among all the values of ${\mathbf{r}}[n],$ we find the maximal value of $\bar{{\mathbf{z}}}[n],$ which is the suboptimal solution of problem (23). Finally, the optimal phase shift for problem (18) is $\psi _l[n]=\text {arg}\big ({\bar{z}}_l[n]/{\bar{z}}_{\text {L}+1}[n]\big ).$ For the active IRS, similar methods can be used to adjust the phase shifts to improve the communication performance.

### UAV trajectory and speed optimization

Given ground sensor’s transmit power ${\mathbf{P}}=\{\text {P}[n],\forall n\},$ the IRS’s passive beamforming $\varvec{\Psi }=\{\varvec{\Psi }[n],\forall n\},$ we will optimize the UAV’s speed and trajectory. Problem (12) is reformulated as

28a $$\begin{aligned} \text{(OP3.1): }&\mathop {\max }\limits _{{\mathbf{Q}},{\mathbf{v}},\varvec{\zeta },\varvec{\eta }} \,\, \frac{\delta _t{\sum _{n=1}^\text {M} \tilde{\text {R}}[n]}}{\text {E}^{\text {total}}}\end{aligned}$$

28b $$\begin{aligned} \text{ s.t.: }&(11\text {b})-(11\text {e}), (12\text {c}), (12\text {d}) \end{aligned}$$

By introducing auxiliary variable $\varvec{\rho }=\{\rho [n],\forall n\},$ the problem (28) is transformed as

29a $$\begin{aligned} \text{(OP3.2): }&\mathop {\max }\limits _{{\mathbf{Q}},{\mathbf{v}},\varvec{\zeta },\varvec{\eta },\varvec{\rho }} \,\, \frac{\delta _t{\sum _{n=1}^\text {M}\log _2(1+\frac{\text {P}[n]\rho [n]}{\sigma ^2})}-\sqrt{\frac{\zeta [n]}{m}}\frac{{Q}^{-1}(\epsilon )}{\ln 2}}{\text {E}^{\text {total}}}\end{aligned}$$

29b $$\begin{aligned} \text{ s.t.: }&(11\text {b})-(11\text {e}), (12\text {c}), (12\text {d}) \end{aligned}$$

29c $$\begin{aligned}&\rho [n]\le \Big |h_{du}[n]+{\mathbf{h}}_{ru}^\text {H}[n]\varvec{\Psi }[n]{\mathbf{h}}_{dr}\Big |^2,\forall n \end{aligned}$$

It is seen that (29a) is nonconcave and the constraints (12c), (12d) and (29c) are nonconvex. Similar to the previous section, we equivalently transform constraint (12c) into constraint (16).

To facilitate the analysis, we give the following equation

30 $$\begin{aligned} \begin{aligned}&\Big |h_{du}[n]+{\mathbf{h}}_{ru}^\text {H}[n]\varvec{\Psi }[n]{\mathbf{h}}_{dr}\Big |^2 \\&=\Big [\text {D}_{du}^{-\beta _{du}/2}[n],\text {D}_{ru}^{-1}[n]\Big ]{\mathbf{W}}[n]\Big [\text {D}_{du}^{-\beta _{du}/2}[n],\text {D}_{ru}^{-1}[n]\Big ]^\text {T}, \end{aligned} \end{aligned}$$

where ${\mathbf{W}}[n]=\alpha {\mathbf{w}}^\text {H}[n]{\mathbf{w}}[n],$ ${\mathbf{w}}[n]=\big [{\tilde{h}}_{du}[n],\tilde{{\mathbf{h}}}_{ru}^\text {H}[n]\varvec{\Psi }[n]{\mathbf{h}}_{dr}\big ].$ The elements of $\tilde{{\mathbf{h}}}_{ru}[n]$ are nonconvex and nonlinear with the position of the UAV. This will make it extremely difficult to construct equivalent convex constraints. Therefore, we approximate $\tilde{{\mathbf{h}}}_{ru}[n]$ in the *k*th iteration by utilizing the UAV’s position in the $(k-1)$th iteration.

By introducing auxiliary variables ${\mathbf{u}}=\{u[n],\forall n\}$ and $\varvec{\chi }=\{\chi [n],\forall n\},$ we equivalently transform constraint (12d) into

31 $$\begin{aligned} \eta [n]\ge \frac{\text {P}[n]{\mathbf{E}}[n]{\mathbf{W}}[n]{\mathbf{E}}^\text {T}[n]}{\sigma ^2}, \end{aligned}$$

32 $$\begin{aligned} u^{-4/\beta _{du}}[n]\le \text {D}_{du}^2[n], \end{aligned}$$

33 $$\begin{aligned} \chi ^{-2}[n]\le \text {D}_{ru}^2[n], \end{aligned}$$

where ${\mathbf{E}}[n]=\big [u[n],\chi [n]\big ]$. Since the right-hand side functions of (32) and (33) are both convex, their concave lower bounds can be obtained as follows

34 $$\begin{aligned} \text {D}_{du}^2[n]\ge x_d^2-2x_dx_u[n]+y_d^2-2y_dy_u[n]+(z_d-z_u)^2+\text {F}[n],\end{aligned}$$

35 $$\begin{aligned} \text {D}_{ru}^2[n]\ge x_r^2-2x_rx_u[n]+y_r^2-2y_ry_u[n]+(z_r-z_u)^2+\text {F}[n], \end{aligned}$$

where

36 $$\begin{aligned} \begin{aligned} \text {F}[n]=2x_{u,0}[n]x_u[n]-x_{u,0}^2[n]+2y_{u,0}[n]y_u[n]-y_{u,0}^2[n]. \end{aligned} \end{aligned}$$

Similar to constraint (12d), we add two auxiliary variables ${\mathbf{e}}=\{e[n],\forall n\}$ and $\varvec{\tau }=\{\tau [n],\forall n\}$ to decompose constraint (29c) into the following three constraints

37 $$\begin{aligned} \rho [n]\le {\mathbf{B}}[n]{\mathbf{W}}[n]{\mathbf{B}}^\text {T}[n], \end{aligned}$$

38 $$\begin{aligned} e^{-4/\beta _{du}}[n]\ge \text {D}_{du}^2[n], \end{aligned}$$

39 $$\begin{aligned} \tau ^{-2}[n]\ge \text {D}_{ru}^2[n], \end{aligned}$$

where ${\mathbf{B}}[n]=\big [e[n],\tau [n]\big ].$ Constraint (37) is nonconvex, however, ${\mathbf{B}}[n]{\mathbf{W}}[n]{\mathbf{B}}^\text {T}[n]$ is convex to ${\mathbf{B}}[n].$ Then, its concave lower bound can be obtained as follows

40 $$\begin{aligned} \begin{aligned} {\mathbf{B}}[n]{\mathbf{W}}[n]{\mathbf{B}}^\text {T}[n] \ge 2{\mathfrak {R}}\big [{\mathbf{B}}_0[n]{\mathbf{W}}[n]{\mathbf{B}}^\text {T}[n]\big ]-{\mathbf{B}}_0[n]{\mathbf{W}}[n]{\mathbf{B}}_0^\text {T}[n]. \end{aligned} \end{aligned}$$

In constraints (38) and (39), $e^{-4/\beta _{du}}[n]$ and $\tau ^{-2}[n]$ are convex to *e*[*n*] and $\tau [n],$ respectively. Then, the constraints (38) and (39) can be replaced by the following constraints

41 $$\begin{aligned} e_0^{-4/\beta _{du}}[n]-4e_0^{-(1+4/\beta _{du})}[n](e[n]-e_0[n])\big /{\beta _{du}} \ge \text {D}_{du}^2[n], \end{aligned}$$

42 $$\begin{aligned} \tau _0^{-2}[n]-2\tau _0^{-3}[n](\tau [n]-\tau _0[n])\ge \text {D}_{ru}^2[n]. \end{aligned}$$

Since problem (28) is a fractional maximization problem, we will perform additional steps to obtain an objective function with a concave-convex structure. Then, problem (28) is solved by using existing fractional programming (FP) methods. Given the point $\zeta _0,$ the concave lower bound of the numerator of the objective function is

43 $$\begin{aligned} \begin{aligned}&\log _2\bigg (1+\frac{\text {P}[n]\rho [n]}{\sigma ^2}\bigg )-\sqrt{\frac{\zeta [n]}{m}}\frac{{Q}^{-1}(\epsilon )}{\ln 2} \\&\ge \log _2\bigg (1+\frac{\text {P}[n]\rho [n]}{\sigma ^2}\bigg )-\Big \{\zeta _0^{1/2}[n]+\zeta [n]\zeta _0^{-1/2}[n]\Big \}\frac{{Q}^{-1}(\epsilon )}{2\sqrt{m}\ln 2}\\&=\tilde{\text {R}}_Q^{lb}[n]. \end{aligned} \end{aligned}$$

With the assistance of the auxiliary variable ${\mathbf{c}}=\{c[n],\forall n\},$ $\text {E}^{\text {total}}$ can be transformed into

44 $$\begin{aligned} \bar{\text {E}}^{\text {total}}=\delta _t\sum _{n=1}^\text {M}\bigg [\frac{1}{2}s_0\Vert {\mathbf{v}}[n]\Vert ^3+\text {P}_1(1+\frac{3\Vert {\mathbf{v}}[n]\Vert ^2}{\varpi ^2})+\text {P}_2c[n]\bigg ], \end{aligned}$$

and the constraint

45 $$\begin{aligned} c^{-2}[n]-c^2[n]\le \frac{\Vert {\mathbf{v}}[n]\Vert ^2}{\text {U}_0^2}. \end{aligned}$$

In (45), $(c^{-2}[n]-c^2[n])$ is a difference convex structure with respect to *c*[*n*], and ${\Vert {\mathbf{v}}[n]\Vert ^2}/{\text {U}_0^2}$ is convex to ${\mathbf{v}}[n].$ Therefore, by using the SCA method, we can rewrite constraint (45) as

46 $$\begin{aligned} \begin{aligned} c^{-2}[n]+c_0^2[n]-2c_0[n]c[n]\le \frac{1}{\text {U}_0^2}\Big [\Vert {\mathbf{v}}_0[n]\Vert ^2+2{\mathbf{v}}_0^\text {T}[n]({\mathbf{v}}[n]-{\mathbf{v}}_0[n])\Big ]. \end{aligned} \end{aligned}$$

Then, the problem (28) is reformulated as follows

47a $$\begin{aligned} \text{(OP3.3): }&\mathop {\max }\limits _{\mathbf{Q},\mathbf{v},\varvec{\zeta },\varvec{\eta },\varvec{\rho },\mathbf{e},\varvec{\tau },\mathbf{u},\varvec{\chi },\mathbf{c}} \,\, \frac{\delta _t{\sum _{n=1}^\text {M}\tilde{\text {R}}_Q^{lb}[n]}}{\bar{\text {E}}^{\text {total}}}\end{aligned}$$

47b $$\begin{aligned} \text{ s.t.: }&(11\text {b})-(11\text {e}), (16), (31), (41), (42), (46) \nonumber \\&u^{-4/\beta _{du}}[n]\le x_d^2-2x_dx_u[n]+y_d^2-2y_dy_u[n]+(z_d-z_u)^2+\text {F}[n],\forall n \nonumber \\&\chi ^{-2}[n]\le x_r^2-2x_rx_u[n]+y_r^2-2y_ry_u[n]+(z_r-z_u)^2+\text {F}[n],\forall n \nonumber \\&\rho [n]\le 2\mathfrak {R}\Big [\mathbf{B}_0[n]\mathbf{W}[n]\mathbf{B}^\text {T}[n]\Big ]-\mathbf{B}_0[n]\mathbf{W}[n]\mathbf{B}_0^\text {T}[n], \forall n \end{aligned}$$

In (47a), ${\delta _t{\sum _{n=1}^\text {M}\tilde{\text {R}}_Q^{lb}[n]}}/{\bar{\text {E}}^{\text {total}}}$ is a fraction with a concave-convex structure, hence Dinkelbach’s method is used to solve Problem (47). According to^44^, we transform Problem (47) by introducing multiplication factor $\iota _k$ as follows

48a $$\begin{aligned} \text{(OP3.4): }&\mathop {\max }\limits _{{\mathbf{Q}},{\mathbf{v}},\varvec{\zeta },\varvec{\eta },\varvec{\rho },{\mathbf{e}},\varvec{\tau },{\mathbf{u}},\varvec{\chi },{\mathbf{c}}} \,\, \delta _t{\sum _{n=1}^\text {M}\tilde{\text {R}}_Q^{lb}[n]}-\iota _k\bar{\text {E}}^{\text {total}}\end{aligned}$$

48b $$\begin{aligned} \text{ s.t.: }&(11\text {b})-(11\text {e}), (16), (31), \nonumber \\&(41), (42), (46), (47\text {c})-(47\text {e}) \end{aligned}$$

where $\iota _k$ denotes the factor value at the *k*th iteration. Problem (48) is convex, so we can use CVX toolbox to solve it.

**Algorithm 1:**
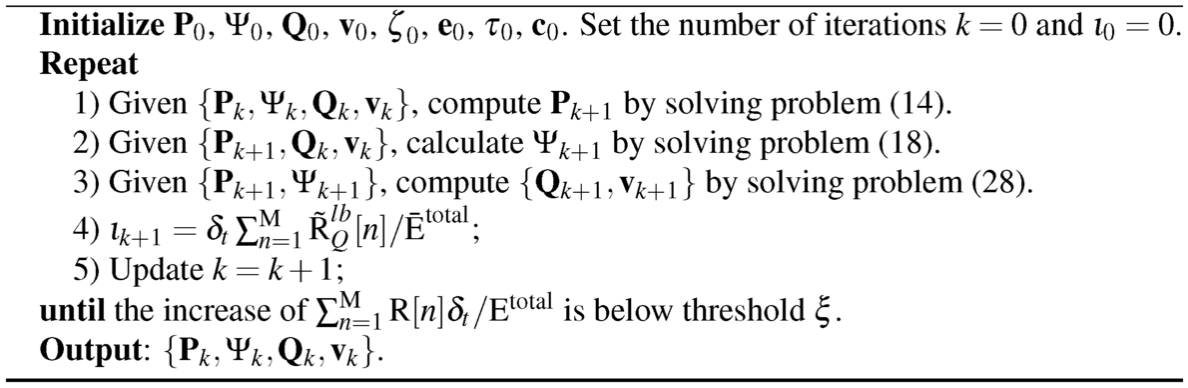
Joint Optimization Algorithm for Problem (11)

### Joint optimization algorithm

Based on the analyses in sections “Transmit power optimization”, “Passive beamforming optimization” and “UAV trajectory and speed optimization”, we have transformed the transmit power optimization, passive beamforming optimization, UAV trajectory and speed optimization problem into standard convex optimization problems. Then, an efficient iterative algorithm will be proposed by optimizing the subproblems (17), (27) and (48) in an iterative manner. The proposed algorithm is shown in Algorithm 1.

According to^45^, the energy efficiency $\Upsilon$ is non-decreasing at each iteration. The convergence of Algorithm 1 is proved as follows.

49 $$\begin{aligned} \begin{aligned}&\Upsilon (\text {P}_k,\varvec{\Psi }_k,\text {Q}_k,\text {v}_k) \\&{\mathop {\le }\limits ^{(a)}}\Upsilon (\text {P}_{k+1},\varvec{\Psi }_k,\text {Q}_k,\text {v}_k)\\&{\mathop {\le }\limits ^{(b)}}\Upsilon (\text {P}_{k+1},\varvec{\Psi }_{k+1},\text {Q}_k,\text {v}_k)\\&{\mathop {\le }\limits ^{(c)}}\Upsilon (\text {P}_{k+1},\varvec{\Psi }_{K+1},\text {Q}_{k+1},\text {v}_{k+1}),\\ \end{aligned} \end{aligned}$$

where the conditions (a), (b) and (c) hold since $\text {P}_{k+1},$ $\varvec{\Psi }_{K+1},$ $\text {Q}_{k+1}$ and $\text {v}_{k+1}$ are the optimal solutions of subproblems (17), (27) and (48), respectively. The maximal number of iterations of Algorithm 1 is supposed to be $k_{\max },$ the parameters $\text {P},$ $\varvec{\Psi },$ $\text {Q}$ and $\text {v}$ are convergent when $\Upsilon$ converge.

The proposed Algorithm 1 has fast convergence speed, which will be shown in the simulations. Next, the complexity of the algorithm will be analyzed. In step 3, the complexity is $\Omega _1={\mathcal {O}}(\omega _1(3\text {M})^{3.5}),$ where $\omega _1$ is the number of iterations in step 3. In step 4, the complexity is $\Omega _2={\mathcal {O}}(\omega _2\sqrt{\text {L}+1}(\text {M}(\text {L}+1)^3+\text {M}^2(\text {L}+1)+\text {M}^3)),$ where $\omega _2$ is the number of iterations in step 4. In step 5, the complexity is $\Omega _3={\mathcal {O}}(\omega _3(12\text {M})^{3.5}),$ where $\omega _3$ is the number of iterations in step 5. Therefore, the complexity of the proposed algorithm 1 is ${\mathcal {O}}(k(\Omega _1+\Omega _2+\Omega _3))$.

## Numerical results

In the simulations, we suppose that the flight altitude of the UAV is $z_u=100$m, the initial position of the UAV is ${\mathbf{Q}}_\text {I}=(-150,-150)$ and the final position is ${\mathbf{Q}}_\text {F}=(150,-150)$. And the horizontal positions of the ground sensor *d* and the IRS are ${\mathbf{C}}_d=(0,50)$ and ${\mathbf{C}}_r=(0,0)$, respectively. The altitude of the ground sensor *d* is $z_d=10$m. The altitude of IRS is $z_r=30$m. The time slot is set to be $\delta _t=\text {T}/\text {N}=1$s. $\beta _{du}=3.8,$ $\beta _{dr}=2.3,$ and the reflecting element spacing of the IRS is set to be $d=\lambda /2.$ The maximal transmit power of ground sensor *d* is $\text {P}_{\max }=1$W. The average transmit power of ground sensor *d* is $\text {P}_{\text {avg}}=\text {P}_{\max }/2$. The value of the threshold in the Joint Optimization Algorithm is $\xi =0.001$. The UAV’s maximum speed is $v_{\max }=40$m/s, and the minimum flight speed of the UAV is $v_{\min }=3$m/s. Other parameter settings are given in Table 1.

**Fig. 2:**
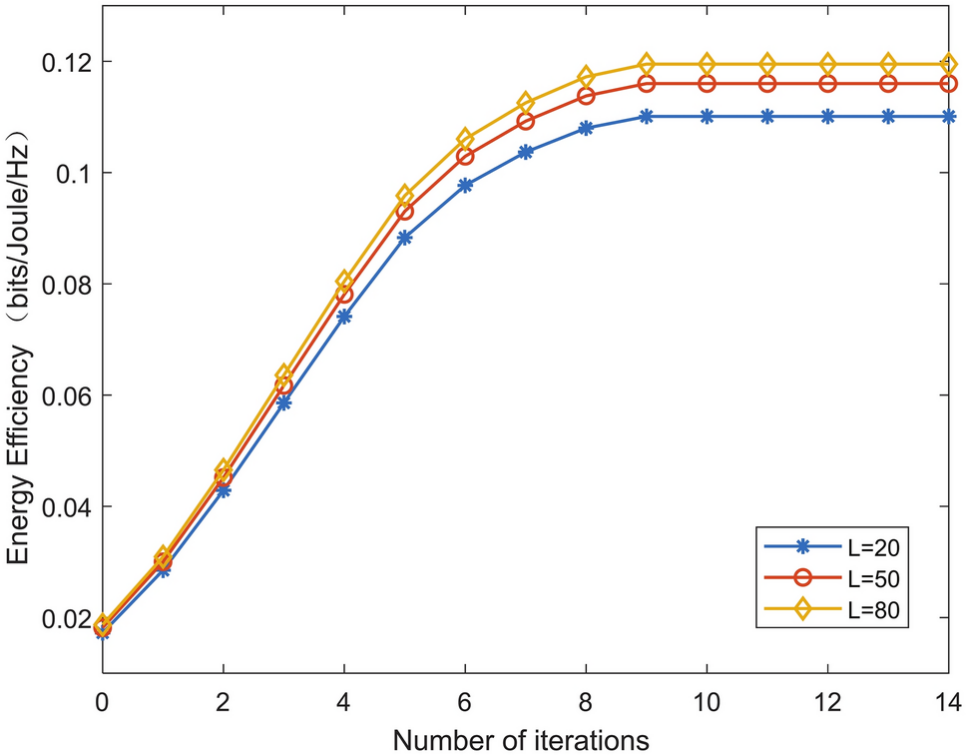
Convergence performance of Algorithm 1.

**Table 1:** Parameter settings.

Parameter	Value
Flight time of the UAV	$\text {T}=60$s
Blocklength of the transmitted packet	$m=500$
Decoding error probability of the system	$\epsilon =10^{-6}$
Variance of the AWGN	$\sigma ^2=-170$dBm
Channel power gain with unit distance	$\alpha =-20$dB
Number of reflecting elements	$\text {L}=20$
Rician fading factor	$\kappa =5$
Power parameters in hovering state of the UAV	$\text {P}_1=178$W, $\text {P}_2=392$W
Parameter related to parasite power of the UAV	$s_0=0.0185$
Parameters related to rotor blade velocity of the UAV	$\varpi =120$, $\text {U}_0=3.57$m/s

Figure 2 shows the performance of convergence of algorithm 1. As seen in Fig. 2, the proposed algorithm can reach convergence after ten iterations. Fig. 2 also shows the relationship between the energy efficiency and the number of reflecting elements L. It is shown that the energy efficiency of the considered system increases with the value of L.

**Fig. 3:**
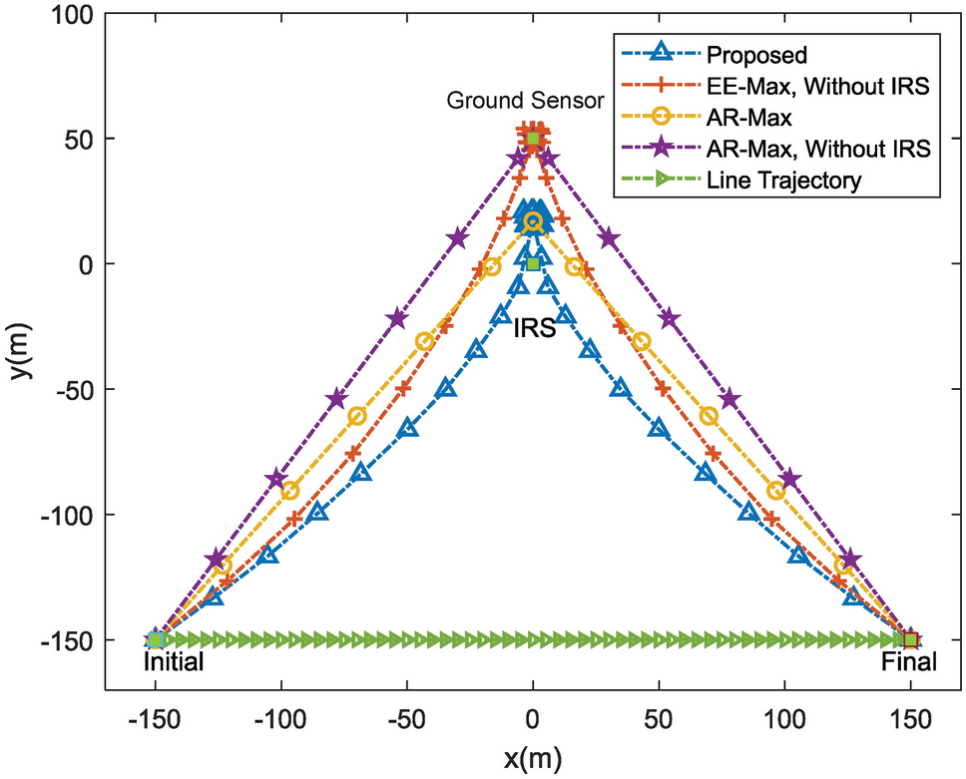
Comparison of UAV trajectories for different schemes.

In Fig. 3, the UAV trajectory of the proposed scheme is compared with four schemes: (1) “EE-Max, without IRS”: In this scheme, we consider maximizing the energy efficiency without IRS. (2) “AR-Max”: The average transmission rate of the UAV system is maximized by optimizing the passive beamforming of IRS, UAV’s trajectory and transmit power. (3) “AR-Max, without IRS”: Maximizing the transmission rate without IRS. (4) “Line trajectory”: The energy efficiency maximized with straight line trajectory and fixed value of speed of the UAV. In Fig. 3, it is seen that the UAV’s optimal position is between the ground sensor and the IRS. When there is no IRS, the UAV’s optimal position is above the ground sensor.

**Fig. 4:**
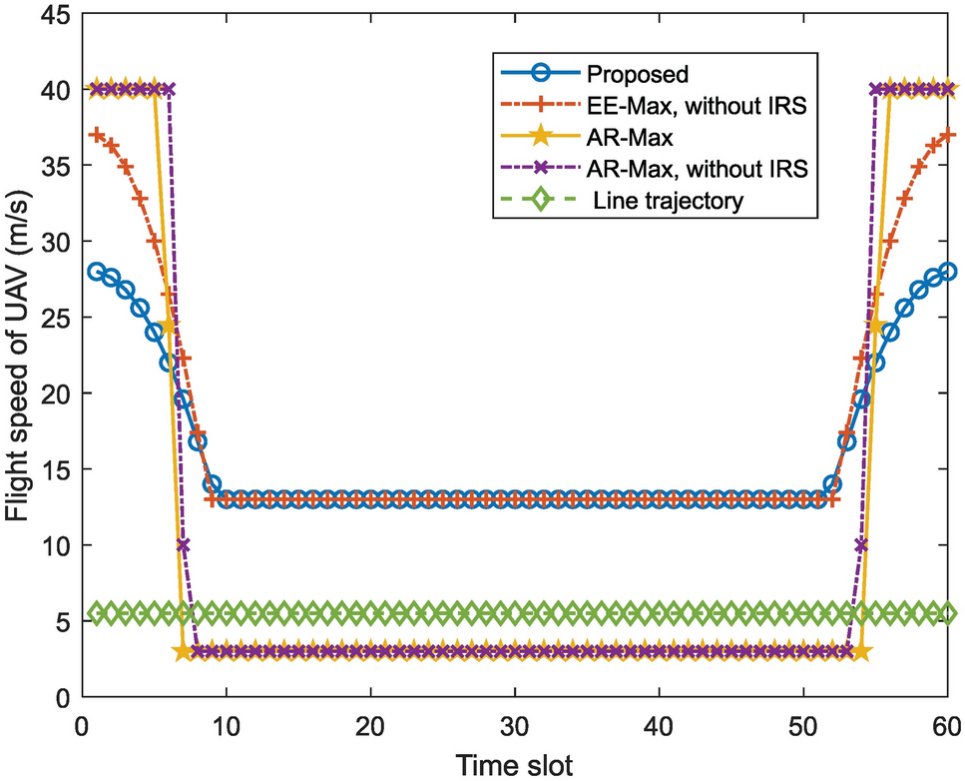
UAV flight speed versus time slot for different schemes.

Figure 4 depicts the UAV flight speed versus time slot for different schemes. The UAV in the “AR-Max” scheme and the “AR-Max, without IRS” scheme will fly to the optimal position with maximum speed, and then circle around at the optimal position with minimum speed. The speed of the UAV decreases gradually in “EE-Max, without IRS” scheme and our proposed scheme, and when the UAV reaches the optimal position, it flies with a fixed speed. This indicates there exists an optimal speed that minimizes the energy consumption.

**Fig. 5:**
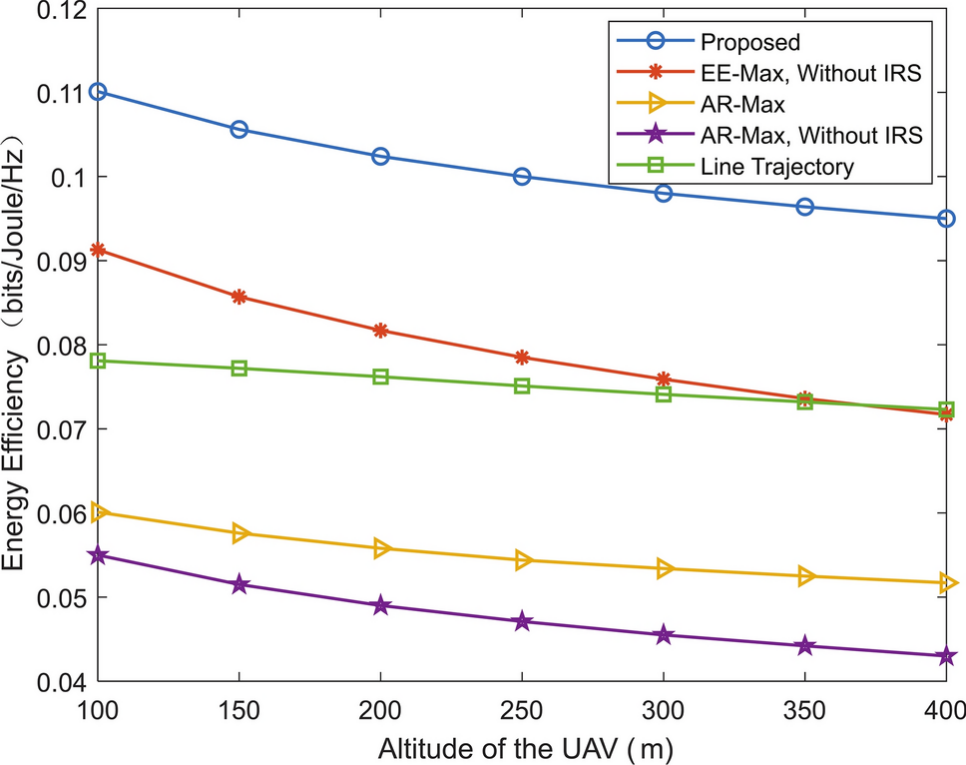
Energy efficiency versus altitude of the UAV for different schemes.

**Fig. 6:**
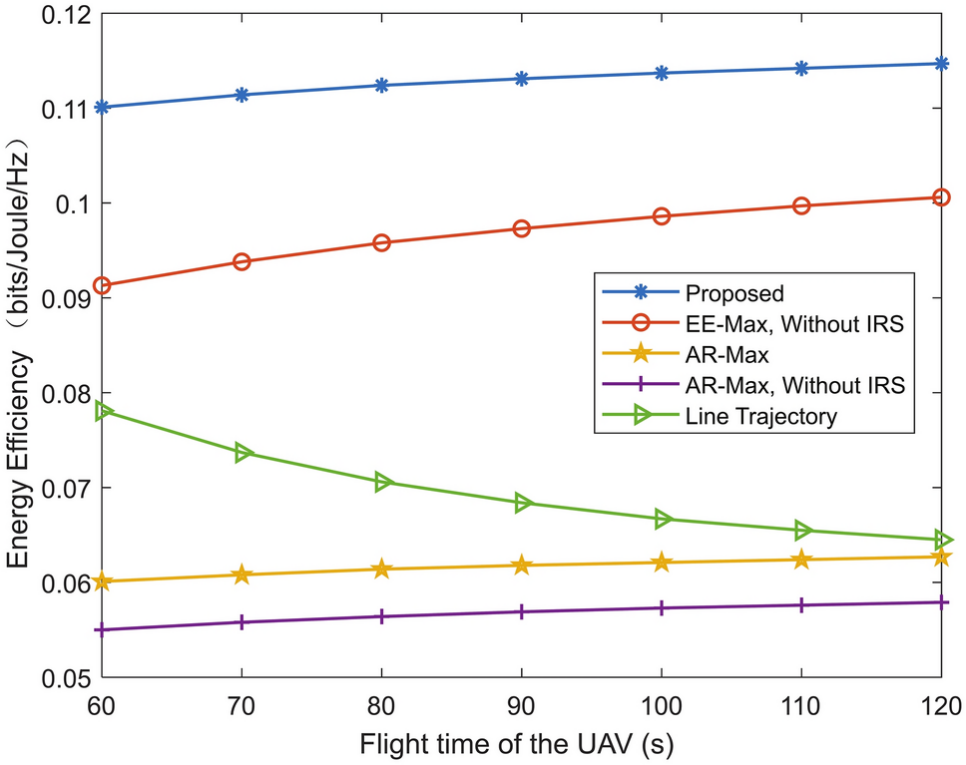
Energy efficiency versus flight time of the UAV for different schemes.

Figure 5 shows the energy efficiency versus altitude of the UAV for different schemes. Compared to other schemes, the proposed scheme can obtain higher value of energy efficiency. By comparing the proposed scheme and “EE-Max, without IRS” scheme, it can be found that the assistance of IRS can enhance the energy efficiency. For example, when the altitude of the UAV is 300m, compared with the “EE-Max, without IRS” scheme, the energy efficiency of the proposed scheme is increased about 25%. Moreover, the proposed scheme and “EE-Max, without IRS” scheme can obtain higher system energy efficiency than “AR-Max” scheme and “AR-Max, without IRS” scheme. This is because both “AR-Max” scheme and “AR-Max, without IRS” scheme are optimized to maximize the transmission rate. And the value of energy efficiency in all considered schemes decreases with altitude of the UAV.

Figure 6 gives the energy efficiency versus flight time of the UAV for different schemes. In the “Line trajectory” scheme, the energy efficiency decreases with the flight time. The reason is that the speed of the UAV decreases with the UAV’s flight time, which results in an increase in the energy consumption. The energy efficiency for the other four schemes increases with the flight time of the UAV. Moreover, the energy efficiency of proposed scheme and “EE-Max, without IRS” scheme increases with the flight time at a faster rate. This is because the two schemes improve the energy efficiency via joint optimization of multiple system parameters.

**Fig. 7:**
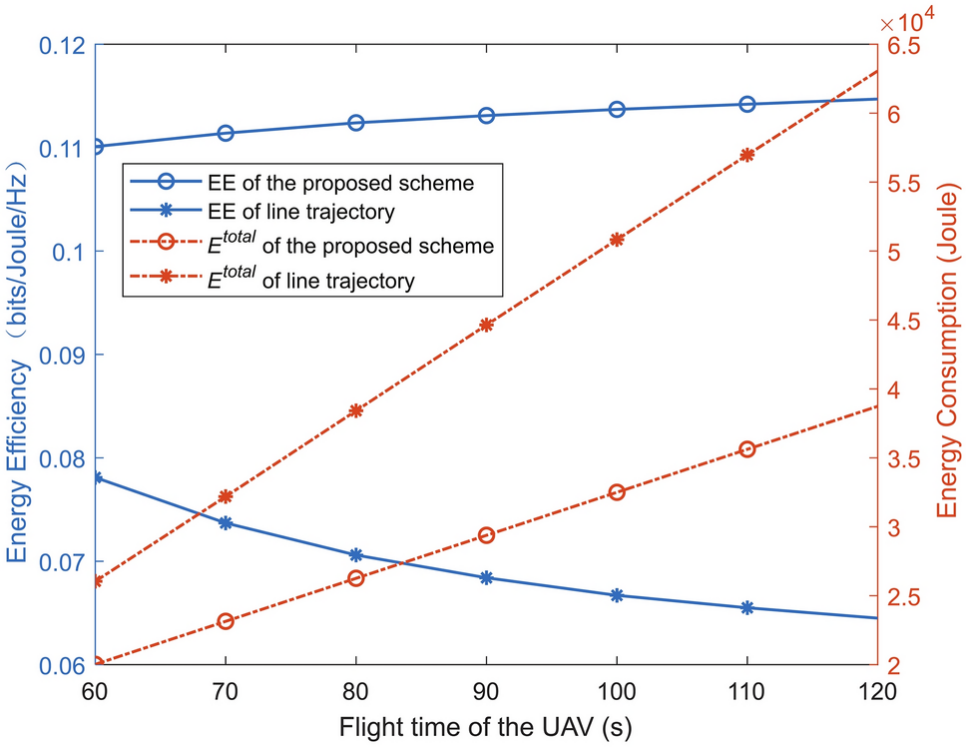
The energy efficiency and energy consumption versus flight time of the UAV.

Figure 7 compares the energy efficiency and energy consumption for different flight times in the “Line trajectory” scheme, where “EE” is the abbreviation for energy efficiency. Compared to “Line trajectory” scheme, the energy consumption of the proposed scheme is significantly reduced by optimizing the speed and trajectory of the UAV. The energy efficiency in the proposed scheme increases with the UAV flight time. The reason is that both the trajectory and speed of the UAV are optimized in the proposed scheme. Thus, the average power consumption of the UAV decreases with the UAV flight time.

**Fig. 8:**
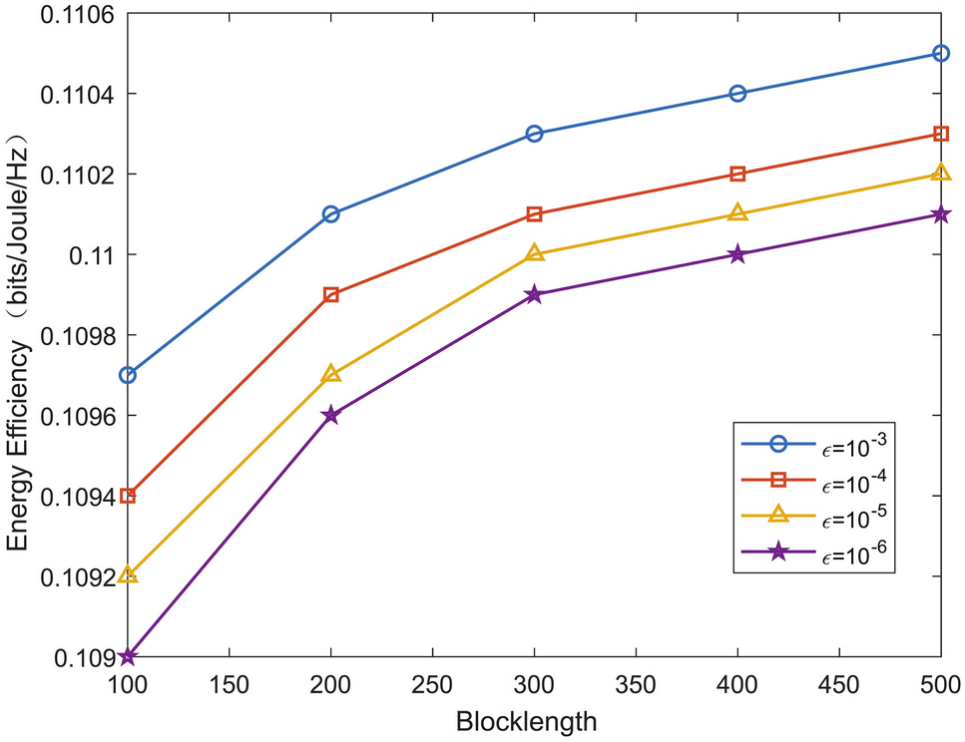
Energy efficiency versus blocklength for various decoding error probabilities.

Figure 8 plots the energy efficiency versus blocklength for various decoding error probabilities. We observe that the energy efficiency increases with blocklength. This is because in short packet communication, the larger the blocklength, the larger the maximum achievable communication rate, which leads to an increase in the energy efficiency. In addition, the energy efficiency of the system is more sensitive to changes in blocklength when the decoding error probability is smaller.

## Conclusions

In this paper, the energy efficiency optimization problem for IRS-assisted UAV short packet communication is studied. We maximize the energy efficiency by jointly designing the transmit power, passive beamforming of IRS, UAV trajectory and speed. The successive convex approximation method and Dinkelbach method are used to solve the optimization problem. Based on the simulation results, we can find that the proposed scheme can achieve higher value of energy efficiency than the benchmark schemes. Compared with the scheme without IRS, it can be found that the application of IRS can improve the energy efficiency effectively.

## Data Availability

The datasets used and analysed during the current study are available from the corresponding author on reasonable request.
